# Recent Advances in Drug Delivery and Oral Health: The Impact of Technology and Digital Advances as a New Frontier

**DOI:** 10.3390/bioengineering12060664

**Published:** 2025-06-17

**Authors:** Cristina Grippaudo, Ludovica Nucci, Marco Farronato

**Affiliations:** 1 UOC di Clinica Odontoiatrica, Dipartimento di Neuroscienze, Organi di senso e Torace, Fondazione Policlinico Universitario A. Gemelli, IRCCS, 00168 Rome, Italy; 2 Dipartimento Universitario Testa Collo ed Organi di Senso, Università Cattolica del Sacro Cuore, 00168 Rome, Italy; 3 Department of Mental and Physical Health and Preventive Medicine, University of Campania Luigi Vanvitelli, 80138 Naples, Italy; ludovica.nucci@unicampania.it; 4 Department of Biomedical, Surgical and Dental Sciences, School of Dentistry, University of Milan, 20100 Milan, Italy; 5 Fondazione IRCCS Cà Granda, Ospedale Maggiore Policlinico, 20122 Milan, Italy

Technological progress is the basis of scientific and clinical advancement in dentistry. Interdisciplinarity plays a crucial role in this technological evolution. It enables the transfer and application of knowledge across various medical fields, including bioengineering, materials science, and data analytics, to address challenges in this field [1,2,3]. The integration of cutting-edge technologies, such as digital imaging, artificial intelligence, and 3D printing, has revolutionized dental practice, from diagnosis to treatment planning and execution. These innovations allow for faster, more accurate diagnoses, more effective treatment strategies, and a higher level of precision in procedures. For instance, digital tools such as cone-beam computed tomography (CBCT) and intraoral scanning have significantly enhanced the ability to visualize and assess complex dental and maxillofacial conditions, leading to more accurate diagnostic results and customized treatment plans [4]. These technologies enhance clinicians’ ability to make informed decisions and have a more personalized approach to dental care, as they allow for individualized treatment plans based on a patient’s unique biological characteristics, medical history, and preferences [5,6]. Moreover, personalized approaches could improve the oral health of each patient. It is globally recognized that oral health is influenced not only by exposure to risk factors but also by an individual’s biological and genetic characteristics. To better understand these relationships, ongoing research is focused on exploring how oral tissues respond to various medications. This research aims to identify both beneficial effects and potential adverse reactions to select the most appropriate treatments for each patient based on their unique genetic makeup and medical history. This approach combines two critical lines of investigation: studying the genetic predispositions of individuals, and examining how these individuals respond to pharmacological interventions. 

In this context, this Special Issue includes several noteworthy articles that contribute to the growing body of knowledge on personalized dental care. Asiri provided a fascinating insight into how computational models can be used to tailor drug choices to the individual patient by evaluating the similarities between different drugs based on patient-specific data. This approach optimizes drug therapy and minimizes the risk of adverse reactions, offering a more personalized approach to dental care [7].

Wu et al. investigated the effects of strontium ranelate (SR) on bone remodeling during orthodontic treatment [8]. As orthodontic therapies are increasingly applied to a broader range of patients, including older individuals, it is critical to understand how the alveolar bone responds to these treatments in conjunction with other medications. Their findings could guide clinical decision-making, particularly in patients receiving orthodontic treatment who are also taking medications for conditions such as osteoporosis or other metabolic bone diseases.

In *“Clinical Performance Evaluation of a Hyaluronic Acid Dental Gel for the Treatment of Traumatic Ulcers in Patients with Fixed Orthodontic Appliances: A Randomized Controlled Trial”*, Tremolati et al. examined the efficacy of a new treatment for a common complication of orthodontic therapy: traumatic ulcers caused by the friction of fixed appliances against the oral mucosa [9]. This research explores the potential of hyaluronic acid as a therapeutic agent for promoting healing and reducing discomfort in patients undergoing orthodontic treatment. These studies highlight the increasing importance of personalized medicine in dentistry, with a focus on understanding how genetic, biological, and pharmacological factors interact to influence treatment outcomes.

Dental research is increasingly grounded in in vitro studies of the hard tissues that form the dentoskeletal unit. Recent technological advances have provided researchers with high-precision tools, enabling more accurate preparations for experimental work. One such advancement is highlighted in the article *“Arduino Automated Microwave Oven for Tissue Decalcification”*: an innovative, automated system designed to enhance the efficiency and consistency of tissue decalcification, a critical step in preparing dentoskeletal samples for analysis [10]. 

In the imaging field, numerous studies utilize CBCTs to precisely measure craniofacial bones in various clinical conditions. This data helps to assess the impact of oral pathologies on craniofacial growth and enhances the prognostic process for treatments. The article *“Cone-Beam Computed Tomographic Assessment of Mandibular Condylar Volume in Different Skeletal Patterns: A Retrospective Study in Adult Patients”* provides valuable insights into how facial typology influences condylar growth [11].

Non-invasive diagnostic approaches are widely used to observe the kinematics of the temporomandibular joint (TMJ). One such method has been employed by Scolaro et al. to comprehensively analyze the effectiveness of kinematic parameters as a tool for screening and diagnosing TMJ conditions [12].

Finally, therapy has also been improved by promising new technologies. In this Special Issue, two articles are dedicated to this topic. *“Pulp–Dentin Complex Regeneration with Cell Transplantation Technique Using Stem Cells Derived from Human Deciduous Teeth: Histological and Immunohistochemical Study in Immunosuppressed Rats”* evaluates the possibility of using stem cells in studies before application on humans [13]. *“Impact of Smoking Habit on Peri-Implant Indicators following Different Therapies: A Systematic Review”* analyzes the efficacy of the clinical pathways carried out after implant therapy in smokers, a condition frequently found among our patients [14]. 

In conclusion, the advent of new technologies and medications opens up exciting research opportunities, enabling clinicians to make more informed decisions when choosing treatment options. This progress allows them to approach patient care with a greater sense of awareness, always keeping the individual needs of the patient at the forefront.
